# OAA AND THE GOAL TO END MALNUTRITION

**DOI:** 10.1093/geroni/igz038.854

**Published:** 2019-11-08

**Authors:** Robert Blancato

**Affiliations:** Matz, Blancato & Associates, Washington, District of Columbia, United States

## Abstract

Home delivered and congregate meals programs are a foundation part of the OAA and are a part of each reauthorization process.

